# Extracellular Vesicle-Induced Differentiation of Neural Stem Progenitor Cells

**DOI:** 10.3390/ijms20153691

**Published:** 2019-07-27

**Authors:** Eleonora Stronati, Roberta Conti, Emanuele Cacci, Silvia Cardarelli, Stefano Biagioni, Giancarlo Poiana

**Affiliations:** 1 Dipartimento di Biologia e Biotecnologie “Charles Darwin”, Sapienza Università di Roma, Piazzale Aldo Moro 5, 00185 Roma, Italy; 2 Department of Biological Science, Southern Methodist University, Dallas, TX 75275, USA

**Keywords:** extracellular vesicles, exosomes, neural stem progenitor cells, astrocytes, basic FGF, EGF

## Abstract

Neural stem progenitor cells (NSPCs) from E13.5 mouse embryos can be maintained in culture under proliferating conditions. Upon growth-factor removal, they may differentiate toward either neuronal or glial phenotypes or both. Exosomes are small extracellular vesicles that are part of the cell secretome; they may contain and deliver both proteins and genetic material and thus play a role in cell–cell communication, guide axonal growth, modulate synaptic activity and regulate peripheral nerve regeneration. In this work, we were interested in determining whether NSPCs and their progeny can produce and secrete extracellular vesicles (EVs) and if their content can affect cell differentiation. Our results indicate that cultured NSPCs produce and secrete EVs both under proliferating conditions and after differentiation. Treatment of proliferating NSPCs with EVs derived from differentiated NSPCs triggers cell differentiation in a dose-dependent manner, as demonstrated by glial- and neuronal-marker expression.

## 1. Introduction

During neural development, neural stem progenitor cells (NSPC) with a radial glia identity produce neurons and glial cells in a spatiotemporally regulated manner. This complex process is driven by both extrinsic and intrinsic signals that eventually determine the type and number of adult neural cells [1,2]. Despite major progress in this field, the molecular mechanisms as well as the extrinsic and intrinsic factors underlying lineage commitment in NSPCs remain only partially understood [3,4,5]. Cells displaying properties of neural stem progenitor cells have been isolated from different regions of embryonic and adult murine central nervous systems and cultured in vitro in the presence of growth factors that maintain NSPCs in a proliferative and undifferentiated state [6,7]; these cells retain some radial glia characteristics and upon growth-factor deprivation, NSPCs can undergo differentiation and give rise to a mixed population of neurons and glial cells [8].

The proper development and functionality of the nervous system relies both on the regulation of intrinsic gene expression and on the intercellular communication among neural cells, which may occur through different mechanisms, one of them being the release of different classes of vesicles into extracellular space. Extracellular vesicles are spheroidal particles with different sizes, biogenesis and release mechanisms; among them are exosomes, small vesicles (50–100 nm in diameter) generated by exocytosis of endosomal organelles, multivesicular bodies, secreted by many cell types and characterized by specific contents of lipids, proteins and nucleic acids strictly related to the cells of origin [9,10]. Some of the membrane proteins and lipids found in exosomes are unique to these vesicles and are commonly used as exosomal markers [11]. Comprehensive reviews of the role played by exosomes in a normal healthy nervous system, both during development and in adults have recently been published [12,13,14]. It is now widely accepted that exosomes represent an important vehicle of intercellular communication that allows parental cells to modify gene and protein expression in recipient cells [10,13,15]. Extracellular vesicles may contribute to cell–cell communication during embryonic development [16,17]. Several studies investigated exosome release from neuronal and glial cells [12,14,18] and many efforts have been done to clarify their role in physiological and pathological states [12,19]. It has recently been reported that exosomes derived from mesenchymal stem cells promote neurogenesis and neurite remodeling [18]. The role that extracellular vesicles may play in the mechanism of communication among stem cells has been pointed out in a recent review, “Exchange of genetic information utilizing persistent bidirectional communication mediated by stem cell EVs could regulate stemness, self-renewal and differentiation in stem cells and their subpopulations” [11]. Regulation of neurogenesis by extracellular vesicles in neurogenic niches has also been suggested [20,21,22].

The aim of our study is to determine whether neural progenitors and their progeny produce and secrete EVs and if the latter play a role in the differentiation of neighboring target cells.

## 2. Results

### 2.1. Neural Stem Progenitor Cells Produce and Secrete EVs

To determine whether NSPCs actively produce and secrete EVs, we cultured NSPCs obtained from 13.5-day-old mouse embryos (see Section 4) in Expansion Medium containing both Epidermal Growth Factor (EGF) and basic Fibroblast Growth Factor (bFGF). In these conditions, 85%–90% of cells are immunopositive to an anti-nestin antibody, indicating that they maintain an undifferentiated phenotype.

**Figure 1:**
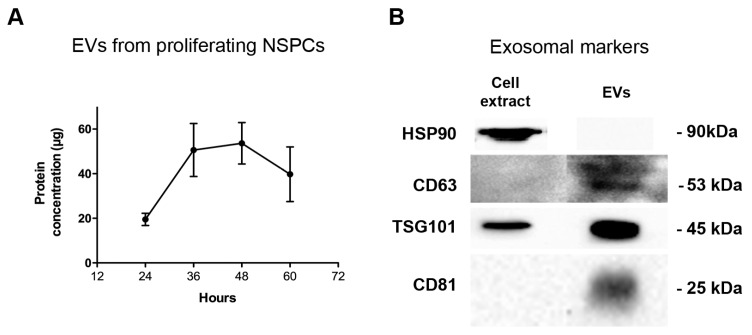
Extracellular vesicle (EV) secretion rate by proliferating neural stem progenitor cells (NSPCs) and analysis with exosomal markers. (**A**) Protein concentration measured in the EV fraction of culture medium of proliferating NSPC. Values are the mean of three independent experiments. (**B**) Western blots of exosomal markers in extracts of proliferating cells and of EVs secreted in 48 h.

We analyzed the culture medium of the proliferating NSPCs and we determined the protein content of the EV fraction obtained as described in Materials and Methods: EVs are actively produced and secreted by proliferating cells and their rate of secretion reaches a maximum between 36 and 48 h in culture when cell confluence is at 60%–70% (Figure 1A).

To ascertain whether the EV fraction from NSPCs cultured in Expansion Medium contained exosomes, we used exosomal markers such as HSP90, CD63, TSG101 and CD81 [23] and analyzed the EV fraction by western blot (Figure 1B), showing that it was enriched in some specific exosomal markers. We did not see any band for HSP90, probably because this specific marker is not present in extracellular vesicles produced by NSPCs. The blot for CD63 was overexposed in order to reveal the signal in the EV fraction.

Secretion was also assessed in NSPC cultures differentiated by using three different protocols. NSPCs were: (a) cultured in the presence of 10 ng/mL bFGF alone (bFGF differentiation medium), obtaining a mixed population of neurons and glial cells; (b) cultured in the presence of 5% Fetal Bovine Serum (FBS differentiation medium); (c) cultured in the presence of 20 ng/mL Bone Morphogenetic Protein 4 (BMP4 differentiation medium) [24]. In the last two culture conditions, enrichment in astroglial cells was achieved. We decided to also use BMP4 as a differentiating agent because, as specified in Section 2.2, its use may prevent the possibility that, even after FBS ultracentrifugation (see Section 4.2), exosomes still present in FBS may have influenced our results.

NSPCs, maintained in the presence of bFGF differentiation medium, differentiated into cells exhibiting an astrocytic and neuronal phenotype, as demonstrated by anti-Glial Fibrillary Acidic Protein (GFAP) and anti-Microtubule Associated Protein (MAP2) labelling. In particular, after three days in culture, 30%–35% cells were GFAP-positive, 15%–20% were positive for the neuronal marker MAP2 and the remaining cells were undifferentiated, as revealed by nestin labelling, in agreement with previous results from our laboratory [8]. When cells were cultured for three days in the presence of either FBS differentiation medium or BMP4 differentiation medium, labelling for GFAP was about 50% and 70% respectively, thus mainly inducing astrocytic differentiation.

**Figure 2:**
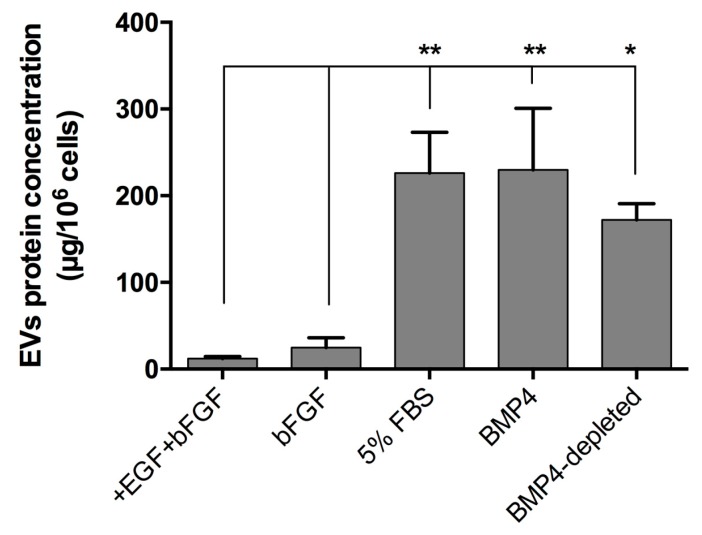
EV secretion by NSPCs maintained under different culture conditions for three days. Proliferating cells were cultured under proliferating conditions (+EGF+bFGF) and in differentiated conditions in the presence of bFGF or FBS or BMP4. Furthermore, cells were maintained for three days with BMP4 and then for two additional days in the absence of BMP4 (BMP4-depleted). Values are the mean of at least three independent experiments. Statistical analysis was performed by using an ANOVA test with multiple comparison. * *p* < 0.05; ** *p* < 0.01.

We compared EV secretion in the medium of NSPCs in proliferating conditions (+EGF+bFGF) and in differentiated NSPCs using three methods to induce cell differentiation, i.e., 10 ng/mL bFGF (bFGF EVs), 5% FBS (FBS EVs) or 20 ng/mL BMP4 (BMP4-EVs). Results are shown in Figure 2.

As shown, cells obtained by astrocytic differentiation released a much higher amount of EVs in extracellular space compared to proliferating cells or differentiated cells obtained omitting EGF. To exclude the effect due to the possible contamination of differentiating factors in the medium of EV collection, we determined the amount of EV released from NSPCs differentiated in the presence of BMP4 for three days and then maintained the cells for two more days in a basal medium. The EVs were collected from the medium of the last two days and referred to as BMP4-depleted EVs. We conclude that cells obtained in the two conditions of astrocytic differentiation released the same amount of EVs and that the amount of EVs was not significantly different, depleting the medium from differentiating agent BMP4 (BMP4 EVS versus BMP4-depleted EVs is not significant, with P = 0.371). Western blot analysis with exosomal markers showed no differences between EVs secreted by proliferating cells and by cells differentiated under the conditions described in Figure 2.

### 2.2. FBS and BMP4 EVs Enhance Differentiation of Proliferating NSPCs

**Figure 3:**
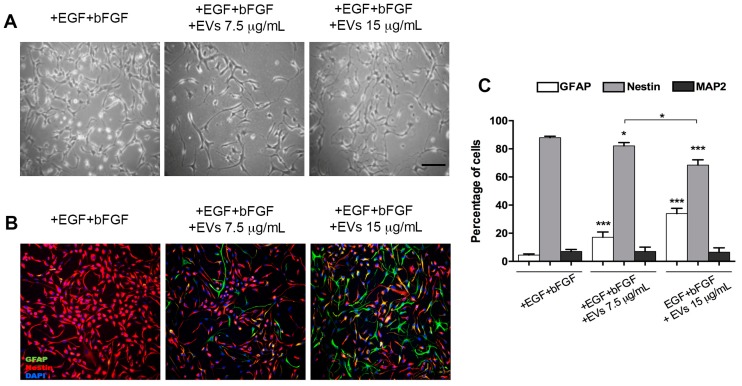
NSPCs maintained in medium containing +EGF+bFGF and treated or untreated with 5% FBS EVs at 7.5 and 15 μg/mL. (**A**) Bright-field images of cultures; (**B**) immunofluorescence labelling with anti-GFAP (green) and anti-nestin (red) antibodies and DAPI (blue); (**C**) quantification of GFAP-, nestin- and MAP2-positive cells after three-day treatment with 5% FBS EVs (7.5 and 15 μg/mL) compared to untreated controls (+EGF+bFGF). Scale bar: 100 μm (images in A and B have the same magnification). Values are mean of at least three independent experiments. * *p* < 0.05; *** *p* < 0.0001.

NSPCs were maintained for three days in Expansion Medium (+bFGF+EGF) and daily supplemented with fresh EV preparations obtained from NSPCs differentiated with FBS differentiation medium. This preparation was called FBS EVs. We used two different concentrations of EVs (7.5 and 15 μg/mL) to assess a dose-dependent effect on these cells due to the EVs. EV concentrations were chosen on the basis of previous observations described in the literature [18,19,25]. After three days of incubation, cells were stained with anti-GFAP, anti-MAP2 and anti-nestin antibodies (Figure 3).

Astroglial-like morphology was developed by cells upon treatment with EVs but it was not exhibited by untreated cells. Percentage of immunostained cells for GFAP was increased, with GFAP-positive cells reaching 18.13% ± 5.11% after EV treatment compared to nontreated NSPCs, with a parallel decrease of nestin expression. No changes in MAP2 staining were observed between treated and untreated NSPCs. Furthermore, we observed that differentiation could depend on EV concentration in the medium. In cells treated with double the concentration of EVs, we observed a higher increase of GFAP-positive cells (34 ± 3.75%) compared to nontreated cells (4.47 ± 0.82%), with a significant additional reduction of nestin-positive cells (from 87.97 ± 0.64% to 68.48 ± 3.47%). In this case, differences in MAP2 staining were also not observed.

Although the centrifugation procedures described in the Materials and Methods section was adopted to avoid the presence of vesicles present in bovine serum [26], residual contamination of morphogenetic or differentiating agents was still possible, so we used the BMP4 differentiation medium to prepare EVs from astrocyte-enriched cultures [24].

Upon BMP4 treatment for three days, the number of cells expressing GFAP increased by up to 70%, giving rise to a higher number of differentiated astrocytes that could produce a more homogeneous EVs population compared with that produced by FBS-differentiated cells.

**Figure 4:**
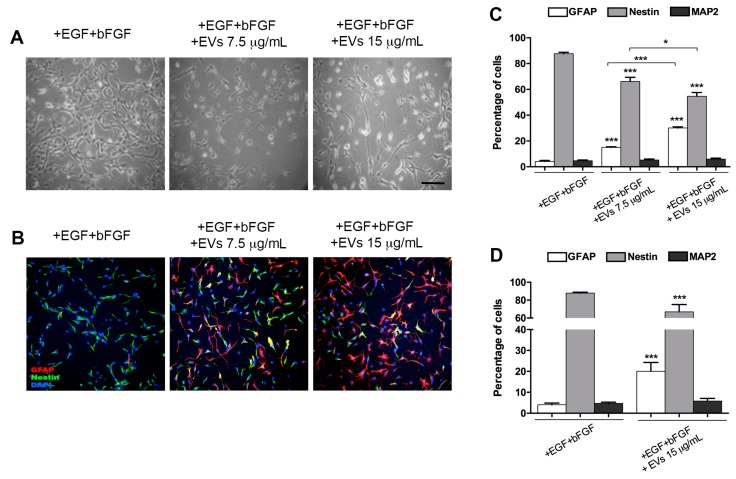
NSPCs maintained in a medium containing +EGF+bFGF, treated or untreated with BMP4 EVs at 7.5 and 15 μg/mL. (**A**) Bright-field images of cultures; (**B**) immunofluorescence labelling with anti-GFAP (red) and anti-nestin (green) antibodies and DAPI (blue); (**C**) quantification of GFAP-, nestin- and MAP2-positive cells, incubated for 72 h with BMP4 EVs (7.5 and 15 μg/mL), obtained from cells treated for three days with BMP4, compared to untreated controls (+EGF+bFGF); (**D**) quantification of GFAP-, nestin- and MAP2-positive cells, incubated for 72 h with EVs (15 μg/mL) obtained from cells, first treated for three days with BMP4 and then for two additional days without BMP4 (BMP4-depleted), compared to untreated controls (+EGF+bFGF). Scale bar: 100 μm (images in **A** and **B** have the same magnification). Values are the mean of at least three independent experiments. * *p* < 0.05; *** *p* < 0.0001.

The treatment of proliferating NSPCs with BMP4 EVs yielded several astrocytes significantly higher than the control cultures and the double concentration produced a significant increase of astrocytes with respect to both controls and to the lower EV concentration (Figure 4A–C).

EVs collected during BMP4 treatment could themselves contain some BMP4 trapped during EV formation, which could itself in turn, induce a differentiating effect on EV-treated cells. Therefore, we treated NSPCs for three days with BMP4, then removed the medium containing BMP4 and collected and used the EVs secreted in the following 48 h in a BMP4-free medium (BMP4-depleted EVs). In this latter case, there was also significant increase in GFAP-positive cells (20.04 ± 4.21%) with respect to controls (4.06 ± 0.81%) and a decrease in nestin-positive undifferentiated cells (Figure 4D).

In all experiments carried out with BMP4 EVs, MAP2-positive cells were always well below 10% of total cells and there was never any difference between the control and treated cells.

## 3. Discussion

In this paper, we showed that NSPCs from embryonic mouse spinal cords can release EVs among which exosomes are present, as demonstrated by the enrichment of well-known exosomal markers (TSG-101, tetraspanins CD63 and CD81) in our samples. The rate of secretion appears constant for about 48 h, although exogenous factors, such as time in culture and cell-to-cell contact could have a significant impact.

Proof that NSPCs-derived EVs play a role in intercellular communication requires that these vesicles are taken up by NSPCs and that this event can modify the biology of target cells. It has been demonstrated that exosomes are uptaken in a selective way by neighboring cells and that their uptake can lead to the activation of cell proliferation and differentiation [10,18,27].

We also demonstrated that EVs produced by differentiated neural progenitor cells can influence the phenotype of target cells; in particular, they can stimulate differentiation of proliferating NSPCs. In addition, we obtained EVs from cells that had been differentiated by two different methods: One pool of EVs was obtained from NSPCs differentiated with 5% FBS (FBS EVs) and the other was produced by NSPCs cultured in the presence of BMP4 (BMP4 EVs); under both these culture conditions, NSPCs differentiated in a highly enriched astrocyte population. Proliferating NSPCs, treated with the EV pools produced in these two ways, showed an induction of differentiation toward an astrocytic lineage. Treatment with FBS EVs gave rise to approximately 40% astrocytes, while the remaining cells were still undifferentiated. Using BMP4 EVs, the yield in astrocytes was higher than controls, with a significant reduction of undifferentiated nestin-positive cells. We also showed that this induction was not dependent on the presence of morphogen BMP4 into the harvested EVs population by using EVs that were from NSPCs differentiated for three days with BMP4 and then maintained for two additional days in the absence of the differentiating agent (BMP4-depleted EVs), after which period EV harvesting took place.

In our experiments, EV treatment seemed to stimulate cells to differentiate, addressing target cells toward an astrocytic phenotype. In the literature, an opposite effect has recently been described, where exosomes produced by C6 glioma cells instead stimulated cell proliferation when incubated with primary cultures of rat cortical astrocytes [25]. The reason for such an opposite effect may reside in the cells of origin of the exosomes: It is likely that C6 glioma cells, being a tumor, may stimulate proliferation in order to increase the number of cells that invade the tissue. It is in fact well established that, together with some general markers that are common to all exosomes regardless of their source, many of the proteins and miRNAs transported by exosomes reflect the nature of their cells of origin.

The original result of this paper is that EVs produced and secreted by normal differentiated embryonic progenitor neural cells may activate the differentiation of normal NSPCs toward a specific phenotype, thus having a potential role in the process that takes place in the neural progenitor cell niche. The results shown in all previous papers reporting similar effects were obtained with exosomes produced either by tumor cells or by cell lines. Our data also suggest that EVs secreted by committed cells can induce or trigger the differentiation of proliferating cells. Furthermore, it is very interesting and worth further investigation that, when NSPCs were treated with EVs obtained from a mixed population of neuronal and glial cells, their differentiation always took place toward an astrocytic phenotype. EVs could contribute to the observed induction toward an astrocytic lineage, possibly by embedding miRNAs that were able to reprogram multiple cellular mechanisms in recipient cells.

Recently, it has been demonstrated that astrocytes and their exosomes share numerous miRNAs but some of them are uniquely expressed in exosomes [28]. It was also proven that vesicles released by cells could reflect the characteristics of their cells of origin (reviewed in [9]). These specific miRNAs present in exosomes probably control some differentiative targets in the recipient cells. In our model, it would be interesting to analyze the vesicular content of FBS or BMP4 EVs in order to clarify their role in the astrocytes’ differentiation induction.

This study demonstrates that EVs from astrocyte-like cells can steer NSPC differentiation toward the glial lineage. We propose that, in addition to contact-dependent signals requiring cell-to-cell connection and the secretion of soluble factors such as cytokines and neurotransmitters from differentiated cells, communication through EVs can also provide the signals required for the activation of the gliogenic process, thereby contributing to the temporarily regulated transition from the neurogenic to the gliogenic phase during development. In the future, it is important to assess whether EV preparations from different neural cell types (e.g., neurons vs. astrocytes) can differently modulate NSPC properties and whether such mechanisms also occur in vivo.

## 4. Materials and Methods

### 4.1. NSPC Cultures

All animal-related procedures were carried out following the European Communities Council Directive No. 86/609/EEC (November 24, 1986). Animals were anesthetized and sacrificed by decapitation.

NSPCs were obtained from E13.5 mouse embryonic spinal cord and adapted to cell cultures as previously described [8,29,30]. Cells were maintained in a medium referred to as the “Expansion Medium” consisting of basal medium (Dulbecco’s Modified Eagle’s Medium (DMEM)/F12, 1% penicillin/streptomycin, 0.1 M L-glutamine (Sigma-Aldrich, St. Louis, MO, USA), 23.8 mg/100 mL Hepes, 7.5% NaHCO3, 0.6% glucose), supplemented with 20 ng/mL human recombinant EGF (EGF; R and D Systems, Minneapolis, MN, USA), 10 ng/mL of basic fibroblast growth factor (bFGF; Peprotec, London, UK) and 1% N2 supplement. NSPCs were routinely expanded in T25 flasks (Corning, Corning, NY, USA), coated with 10 µg/mL polyornithine and 5 µg/mL laminin (Sigma-Aldrich) and cultured at 37 °C in a 5% CO2 atmosphere. Cells were passaged every 3–5 days using Accutase (Sigma-Aldrich) and usually seeded at a density of 10,000–15,000 cells/cm^2^.

### 4.2. Differentiation Conditions

For astrocyte differentiation, NSPCs were plated in Expansion Medium (6000 cells/cm^2^ in T75-coated flasks) and 24 h later the medium was replaced with basal medium containing 1% N2, 2% B27 and 20 ng/mL BMP4 (Peprotech) or 5% FBS (Sigma-Aldrich) and termed differentiating media. To eliminate the risk of adding EVs possibly present in bovine serum, FBS was routinely centrifuged overnight at 100,000 *g* before use [26].

After 3 days in the presence of BMP4, NSPCs differentiated into a highly enriched astrocyte culture (70%–80%); after 3 days of FBS, NSPCs differentiated into a mixed culture of glial cells (about 50%) and residual progenitor cells.

Mixed cultures of neurons and glial cells were obtained omitting EGF from the expansion medium, termed bFGF differentiation medium [31].

### 4.3. Immunocytochemistry

Cells were plated on glass coverslips coated with polyornithine/-laminin. At the appropriate time points, after fixation with 4% paraformaldehyde (PFA, Sigma-Aldrich) in PBS for 20 min, cultures were washed with PBS, pH 7.4 and incubated in PBS containing 5% of the appropriate normal serum and 0.025% Triton X-100 (blocking solution) for 45 min at room temperature. Primary antibodies (monoclonal anti-nestin, Millipore, Burlington, MA, USA) MAB533 1:1000; polyclonal anti-MAP2 Millipore AB5622 1:300; polyclonal anti-GFAP, Dako Z0334 1:500) were then applied and incubated overnight at 4 °C. Cells were then washed 3 times with PBS and incubated for 2 h at room temperature in a solution containing 10 μg/mL Hoechst 33342 and the appropriate secondary antibodies, FITC- or Cy3-conjugated (Jackson Immuno-Research, West Grove, PA, USA) at a dilution of 1:300. After rinsing with PBS, cells were coverslipped with a DAKO mounting medium (Dako, Carpinteria, CA, USA). Immunostaining specificity was assessed by omitting primary antibodies. For each experiment, 9 random fields for each coverslip were examined and 3 or more independent experiments were analyzed. Immunofluorescence was observed using a Nikon Eclipse TE300 microscope. Nine random fields from each coverslip were photographed with a Nikon DS-U3 digital camera and counted. Nuclear DNA was counterstained with Hoechst 33342. At least 400 cells per experiment were counted and each experiment was repeated from 3 or more separate cultures. Results are expressed as mean ± SEM.

### 4.4. Isolation of EVs

Extracellular vesicles were isolated from the NSPC culture medium by sequential ultracentrifugation. Cells were plated at 6000 cells/cm^2^ in 3 T75-coated flasks in expansion medium and 24 h later the medium was replaced with an EV-harvesting medium (basal medium supplemented with 1% Invitrogen supplement (ITS), 5% EV-depleted FBS or 20 ng/mL BMP4). Then, 15 mL of EV-harvesting medium was added to flasks every 36 h, collecting a final volume of 45 mL after 3 days of NSPC differentiation. The medium was subjected to sequential centrifugation and ultracentrifugation (20,000 *g* for 20 min, 10,000 *g* for 30 min and 100,000 *g* for 4 h at 4 °C by using a Sorvall SW40 centrifuge (Thermo Fisher). The obtained EV pellet was resuspended in 1:700 of its initial volume [32] in phosphate buffered saline (PBS) or sample buffer (without beta-mercaptoethanol). Protein concentration was determined by Lowry assay and equal amounts of protein samples were subjected to SDS-PAGE, followed by immunoblotting.

### 4.5. Western Blot Characterization of EVs

To obtain whole-cell extracts, NSPCs were harvested in a RIPA buffer (320 mM sucrose, 50 mM TRIS pH 7.5, 1% Triton X-100, 10% glycerol) and 1% of inhibitor of proteases cocktail (Sigma-Aldrich), incubated on ice for 30 min and centrifuged for 12 min at 13,000 g. Protein amount was quantified by Bradford assay (BioRad, Hercules, CA, USA) and 10–20 μg of protein was subjected to SDS-polyacrylamide gel electrophoresis and transferred overnight onto a polyvinylidene difluoride membrane (Immobilon-PSQ, Millipore). Membranes were blocked in 5% nonfat dry milk and then incubated with the following primary antibodies: rabbit anti-HSP90 (1:300, Santa Cruz Biotechnology, Dallas, TX, USA), mouse anti-TSG101 (1:200, Santa Cruz Biotechnology, Dallas, TX, USA), goat anti-CD63 (1:1000, BD Biosciences, San Jose, CA, USA), mouse anti-CD81 (1:500, BD Biosciences, San Jose, CA, USA). Blots were washed, incubated with the appropriate horseradish peroxidase-conjugated secondary antibodies (1:10,000, Jackson Immunoresearch, West Grove, PA, USA), then washed again and finally incubated in lumilight-enhanced chemiluminescence substrate (BioRad) and exposed to Chemidoc (BioRad). Densitometric analysis on scanned blots was performed using the ImageLab program (BioRad).

### 4.6. Statistical Analysis

Comparisons were performed using Student’s *t*-test or ANOVA test with multiple comparison. Data are the average of 3 or more independent experiments and are presented as mean ± SEM. Differences are considered significant at *p* < 0.05.

## Abbreviations

NSPCs	Neural Stem Progenitor Cells
EGF	Epidermal Growth Factor
bFGF	basic Fibroblast Growth Factor
BMP4	Bone Morphogenetic Protein 4
GFAP	Glial Fibrillary Acidic Protein
MAP2	Microtubule Associated Protein
DMEM	Dulbecco’s Modified Eagle’s Medium
